# The lymphocyte secretome from young adults enhances skeletal muscle proliferation and migration, but effects are attenuated in the secretome of older adults

**DOI:** 10.14814/phy2.12518

**Published:** 2015-11-24

**Authors:** Sarah Al-Dabbagh, Jamie S McPhee, Christopher Murgatroyd, Gillian Butler-Browne, Claire E Stewart, Nasser Al-Shanti

**Affiliations:** 1 Healthcare Science Research Centre, Faculty of Science and Engineering, Manchester Metropolitan UniversityManchester, UK; 2 Myology Center of Research, UM76 - UPMC Sorbonne Universités/U974 - Inserm/FRE3617 - CNRS/AIMParis Cedex 13, France; 3 Research Institute for Sport & Exercise Sciences, School of Sport and Exercise Sciences, Liverpool John Moores UniversityLiverpool, UK

**Keywords:** Differentiation, immune system, lymphocytes, Myoblasts, proliferation, secretome

## Abstract

Older people experience skeletal muscle wasting, in part due to impaired proliferative capacity of quiescent skeletal muscle satellite cells which can be reversed by exposure to young blood. To investigate the role of immune cells in muscle regeneration, we isolated lymphocytes from whole blood of young and older healthy volunteers and cultured them with, or without, anti-CD3/CD28 activators to induce release of cytokines, interleukins, and growth factors into the media. The secreted proteins were collected to prepare a conditioned media, which was subsequently used to culture C2C12 myoblasts. The conditioned media from the activated young lymphocytes increased the rate of proliferation of myoblasts by around threefold (*P* < 0.005) and caused an approximate fourfold (*P* < 0.005) increase in migration compared with nonactivated lymphocyte control media. These responses were characterized by minimal myotube formation (2%), low fusion index (5%), low myosin heavy chain content, and substantial migration. In contrast, myoblasts treated with conditioned media from activated old lymphocytes exhibited a high degree of differentiation, and multi-nucleated myotube formation that was comparable to control conditions, thus showing no effect on proliferation or migration of myoblasts. These results indicate that secreted proteins from lymphocytes of young people enhance the muscle cell proliferation and migration, whereas secreted proteins from lymphocytes of older people may contribute to the attenuated skeletal muscle satellite cell proliferation and migration.

## Introduction

Skeletal muscle satellite cells residing between the basal lamina and sarcolemma in a quiescent state are activated in response to muscle damage or trauma to proliferate, migrate, and differentiate to promote growth and regeneration of fibers (Tidball and Villalta 2010). The progressive loss of skeletal muscle mass and strength characteristic of old age is associated with a failure to appropriately activate the satellite cells (Goldspink et al. 1994; Corsetti et al. 2008), impaired proliferative capacity to produce enough myoblasts essential for muscle regeneration (Conboy et al. 2003), and slowed rates of migration (Price et al. 2007). Consequently, contraction- or trauma-induced muscle damage accumulates while aging through cycles of insufficient regeneration (Conboy et al. 2005; Dumke and Lees 2011).

The failure to activate and proliferate satellite cells in older age is not necessarily only related to intrinsic deficits, but the microenvironment is also implicated (Dumke and Lees 2011; Barberi et al. 2013). For example, skeletal muscle grafted from a young rat and transplanted into an old host showed impaired regeneration, but conversely muscle tissue grafted from an old rat showed extensive regeneration after transplantation into a young host (Carlson and Faulkner 1989). In a Parabiosis model, skeletal muscles of older mice regenerated after the circulation was shared with younger mice (Conboy et al. 2005). In addition, young satellite cells cultured with serum from old mice showed impaired proliferation, while old satellite cells cultured with young plasma proliferated normally (Conboy et al. 2005). These findings suggest that circulating factors, inherent in aging, negatively impact satellite cell activation and muscle regeneration.

Although these data are compelling, controversy exists in relation to human models. For example, when cultured human muscle cells were incubated with plasma derived from young or older adults, there were no significant differences as a consequence of age on proliferation or differentiation of muscle cells (George et al. 2010). It is possible that the small quantities of plasma taken from old people for use during culture with satellite cells did not contain sufficient concentrations of effectors to impact the proliferation, migration, or differentiation. Another concern in terms of using young blood as a rejuvenation strategy is that the young blood may chronically activate stem cells, which would be metabolically demanding and may exhaust the stem cell populations (Scudellari 2015). A more successful model would allow for the activity of the effector proteins to be switched on or off “as needed”. Immune cells, or their secretome, may hold that potential.

In the last decade, there has been a remarkable increase in the number of studies investigating the role of the immune system in muscle regeneration (Aurora and Olson 2014). Immune cells play a potent role in muscle regeneration in vivo (Tidball and Villalta 2010; Burzyn et al. 2013), with data suggesting that satellite cells require the interaction with a number of nonmyogenic cell populations to enable regeneration following damage (Cerletti et al. 2008). For example, the immune system responds to muscle injury with a complex sequence of reactions leading to local inflammation (Tedesco et al. 2010). This inflammatory response, which occurs rapidly after damage, results in the local release of numerous cytokines, interleukins, and growth factors. The infiltrating immune cells and their secretome function to clear the injury site of debris, to attract other cells to the wound site, and to facilitate muscle regeneration – thus suggesting a core role for the immune system in muscle repair in vivo (Burzyn et al. 2013).

Recently, we reported that the proteins secreted by activated lymphocytes, collected from young adults, elicit marked regulatory effects on skeletal muscle proliferation and differentiation; enhancing the former and postponing the latter (Al-Shanti et al. 2014). Thus, we hypothesized that a conditioned media prepared from lymphocytes isolated from young adults would improve skeletal muscle satellite cell proliferation and migration, while effects of lymphocytes from older adults would be attenuated. The purpose of the study was to investigate the effects of the secretome from freshly isolated peripheral blood mononuclear cells (PBMC) derived from young and older men on the rates of proliferation, differentiation, and migration of myoblasts.

## Materials and Methods

### Isolation and culture of human Lymphocytes

The Local Research Ethics Committee approved the study and all participants provided informed written consent. Fresh venous blood was collected from young healthy volunteers (*n* = 20, three female and 17 male, aged 18–25 years) and older volunteers (*n* = 18, two female and 16 male, aged 78–85 years). All participants were healthy, independent living, without mobility limitations, with no recent history of any muscle disease or systemic conditions that affect muscle functions or bone marrow activity, or immunosuppressant or corticosteroids treatment that affect immune function. Lymphocytes were isolated using lymphocytes specific Ficoll–Paque PLUS (GE healthcare Life Science, Buckinghamshire, UK). Prior to culturing the lymphocytes, macrophages were depleted from the cell preparation by preplating for 15 min at 37°C (Karanfilov et al. 1999). To mimic in vivo activation, isolated lymphocytes were cultured at 1 × 10^6^ cell/mL in the absence or presence of antihuman CD3 (OKT3, 10 ng/mL) precoated 96 well flat-bottomed plates as described previously (Garland et al. 2002). Cells were cultured at 37°C, 5% CO_2_ for 4 days in RPMI-1640 containing 10% (v/v) AB human serum, 1% Penicillin/Streptomycin, and 50 U/mL human recombinant interleukin-2 (rhIL-2, R & D System, Abingdon, UK) in the absence or presence of 5 *μ*g/mL anti-CD28 (BD Pharmingen™, San Diego, CA) (Al-Shanti et al. 2014).

### Recovery of secreted cytokines

The lymphocytes culture-media supernatants were recovered and purified using Ultracel-3 membrane tube (Millipore, Watford, UK), which is hereon referred to as the Secretome. Briefly, the secretome was harvested for each treatment group separately (activated and nonactivated; young and elderly) and filtered by centrifugation, using a membrane tube (Ultracel-3 membrane) for 30 min at 5400× g. The harvested secretomes were used in the preparation of conditioned media (CM).

### Conditioned media (CM) preparation

Four different CM were prepared by mixing secretomes with DMEM (one in four parts) which is supplemented with 2% horse serum, 1% Penicillin/Streptomycin, and 200 nmol/L of L-glutamine (standard muscle cell differentiation medium, as follows: young and old CM1, containing secretomes obtained from activated young and old anti-CD3/CD28-lymphocytes, respectively. Three control conditions were also prepared: young and old CM2, containing secretomes obtained from young and old nonactivated lymphocytes (negative control); young and old CM3, containing the secretome from young and old activated lymphocytes, but the secretomes were boiled prior to preparing the conditioned media; CM4, containing the RPMI-1640 with IL-2 and anti-CD3, but without the lymphocytes (Al-Shanti et al. 2014).

### Culture of C2C12 myoblasts

C2C12 murine skeletal myoblasts are a subclone of C2 myoblasts (Yaffe and Saxel 1977) which spontaneously differentiate in culture after serum removal (Blau et al. 1983). The C2C12 cells were purchased from ATCC (Rockville, MD) and maintained by growing the cells at a density of 2×10^6^ cell/flask in 0.2% pregelatinised T75 flasks in a humidified 5% CO_2_ at 37°C. The cells were grown in DMEM (Sigma, Gillingham, Dorset, UK), supplemented with 10% Fetal bovine saline (hi FBS; Thermo Fisher, UK), new born calf serum (hiNCS; Invitrogen, GIBCO, Paisley, UK), 1% L-Glutamate, and 1% of 10,000 U Penicillin/Streptomycin.

### C2C12 treatment with conditioned media

Six-well plates were precoated with 0.2% gelatin for 5 min at room temperature. C2C12 were seeded at 5×10^5^ cell/mL in GM. On attaining 60% confluency, cells were washed twice with sterile PBS. Separate C2C12 cultures were established for young and old CM1 and CM2 (activated and nonactivated lymphocyte secretomes, respectively), CM3 (the lymphocyte secretome was denatured prior to use) and CM4 (culture media without lymphocyte secretome) for 4 days. Morphological differentiation assessments, cell cycle analysis, and migration studies were performed.

### Morphological differentiation and Quantification of the differentiation parameters

Bright-field microscopic analysis was used to assess the phenotypic differentiation (alignment, elongation, and fusion) and immunofluorescence microscopic analysis was used to quantify the differentiation parameters. After 4 days, the C2C12 cultures were fixed in 3.7% formaldehyde solution and incubated at room temperature for 5 min. Cells were washed three times with PBS before being permeabilized with 0.1% Triton X-100 for 5 min. Following Triton removal, cells were either stained with Texas Red®-X Phalloidin (200 unit/mL, Sigma Aldrich, Pool, UK) or AntiMyosin Heavy Chain (MyHC) Alexa Fluor® 488 (eBioscience, Hatfield, UK) and DAPI nuclear counterstain solution (5 mg/mL, IHC WORLD, Woodstock, MD). After 30-min incubation, the treated cells were observed, using a fluorescence microscope. The markers of differentiation were assessed using ImageJ software (Schneider et al. 2012) to evaluate the morphometric parameters of myotube development. The differentiation parameters included: fusion index (FI): calculated by dividing the total number of nuclei per myotube (>2 nuclei) to the total number of nuclei counted over the field x100; the total myotube area (MA): calculated by counting the total area of myotubes in a field over the entire image ×100 (Ren et al. 2008; Ricotti et al. 2011). The aspect ratio (AR) was calculated by dividing the length of myotubes to their width ×100 (Grubišić et al. 2014). Ten random microscopic fields were scanned for each parameter at 10× magnification.

### Cell cycle analysis

Cell cycle analysis was performed as described previously (Al-Shanti and Stewart 2012). After 4 days of culture, C2C12 cells were washed twice with PBS, trypsinized, and fixed with 75% Ethanol at −20°C. After 24 h, cells were washed with PBS and suspended in propidium iodide labeling buffer (50 mg/mL propidium iodide, 0.1 sodium citrate, 20 mg/mL ribonuclease A, and 0.3 Nonidet p-40). The cell cycle events were analyzed, using Cell Quest software from FACS Calibur flow cytometer (BD, Oxford, UK) and cell phase events were identified by ModFit LT v3.0 software.

### Migration study

C2C12 cells were grown in sixwell plates in growth media until 80% confluency. Cell monolayers were washed twice with PBS and incubated for 20 h in DMEM, supplemented with 0.1% HS to attain a quiescent state. After removing the medium, cells were preincubated with Mitomycin C (10 *μ*g/mL, Sigma-Aldrich) (Gamell et al. 2008; Rogel et al. 2011) for 3 h (Gamell et al. 2008) to arrest the proliferative phase. C2C12 cell monolayers were washed three times with quiescence medium and scratched, using a sterile 200 *μ*L tip. The cells were washed three times to remove dead cells and debris. They were cultured in various experimental conditioned media for 18 h at 37°C and 5% CO_2_. Images were obtained at 0 and 18 h of migration, using a Leica DMI6000B microscope (Milton Keynes, UK), ensuring that the same *X* and *Y* coordinates were imaged each time. The number of cells migrated toward the central area, where the cells were initially evacuated following the scratch, was quantified using ImageJ for each treatment (Dimchev et al. 2013).

### Statistical analysis

All experiments were repeated three times independently in duplicate, unless otherwise stated, and were analyzed, using GraphPad Prism software version 5.0 (La Jolla, CA). One-way ANOVA was used to compare the effects of all the experimental conditions followed by Bonferroni post hoc analysis. Results were presented as standard deviation (±SD) of the mean and the significance accepted as *P* < 0.05.

## Results

### Morphological differences in the C2C12 myoblasts treated with CM1 of young and old adults

**Figure 1:**
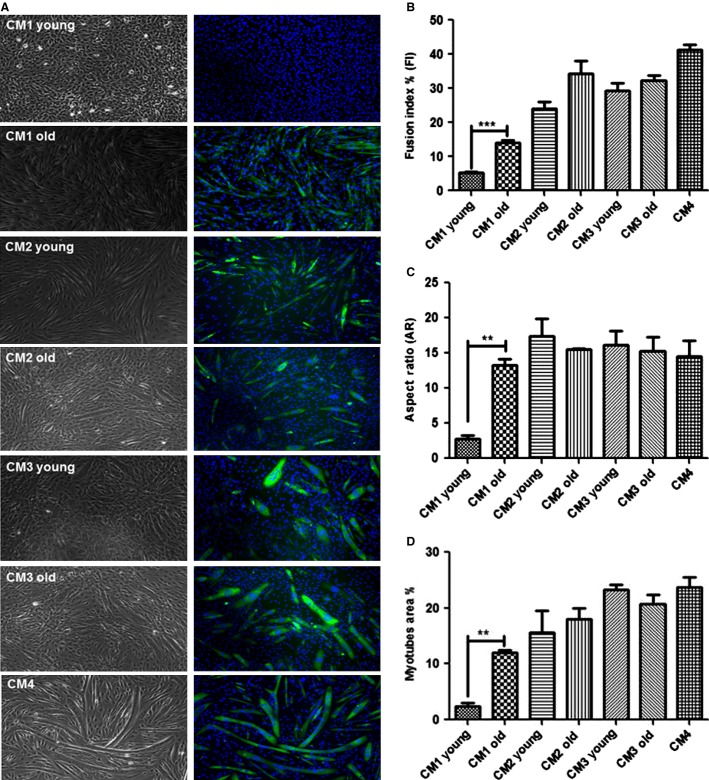
Brightfield (right panel) and Immunofluorescence analysis of skeletal myosin heavy chain (left panel), the typical marker of terminally differentiated muscle cells. Morphometric parameters for myotubes development Myoblast fusion of C2C12 cells following 4 days incubation with CM young and old. C2C12 cell cultures stained immunofluorescence of a high-affinity MHC (marker of differentiation) -heavy-chain Alexa Fluor-488-MyHC (green) and DAPI (blue) nuclear counterstain. All negative controls develop myoblasts fusion into myotubes following 4 days of incubation. Images were taken from random fields by Leica DMI6000B microscope at ×10 magnification. **P < 0.001; ***P < 0.0001.

To evaluate the degree of differentiation and proliferation, immunofluorescence of a high-affinity Alexa Fluor-488-MyHC and DAPI nuclear counterstain were performed (Fig.1A). Analysis of differentiation parameters (Fusion Index, Aspect ratio, and Myotube Area, Fig.1B, C and D) showed that young CM1-treated myoblasts exhibited less differentiation and increased rates of proliferation compared with the old CM1-treated myoblasts. The old CM1-treated myoblasts exhibited significantly more myotube than young CM1-treated myoblasts. This was evident as %FI was lower in young CM1-treated cells compared with old CM1-treated cells (5 ± 0.7% vs. 14.7 ± 1.6%; respectively, *P* < 0.001) and as there was fewer myotubes in young CM1 treatment compared with old CM1 treatment as shown by very low %MA (2.4 ± 0.9% vs. 12 ± 0.8%; respectively, *P* < 0.001). The AR was significantly higher for myotubes in old CM1 compared with young CM1 (13.3 ± 1.3, 2.8 ± 8.7, respectively; *P* < 0.05). There was no significant difference between young and old for the control conditions CM2, CM3, and CM4 for any measurements of proliferation or differentiation.

### Cell cycle analysis

**Figure 2:**
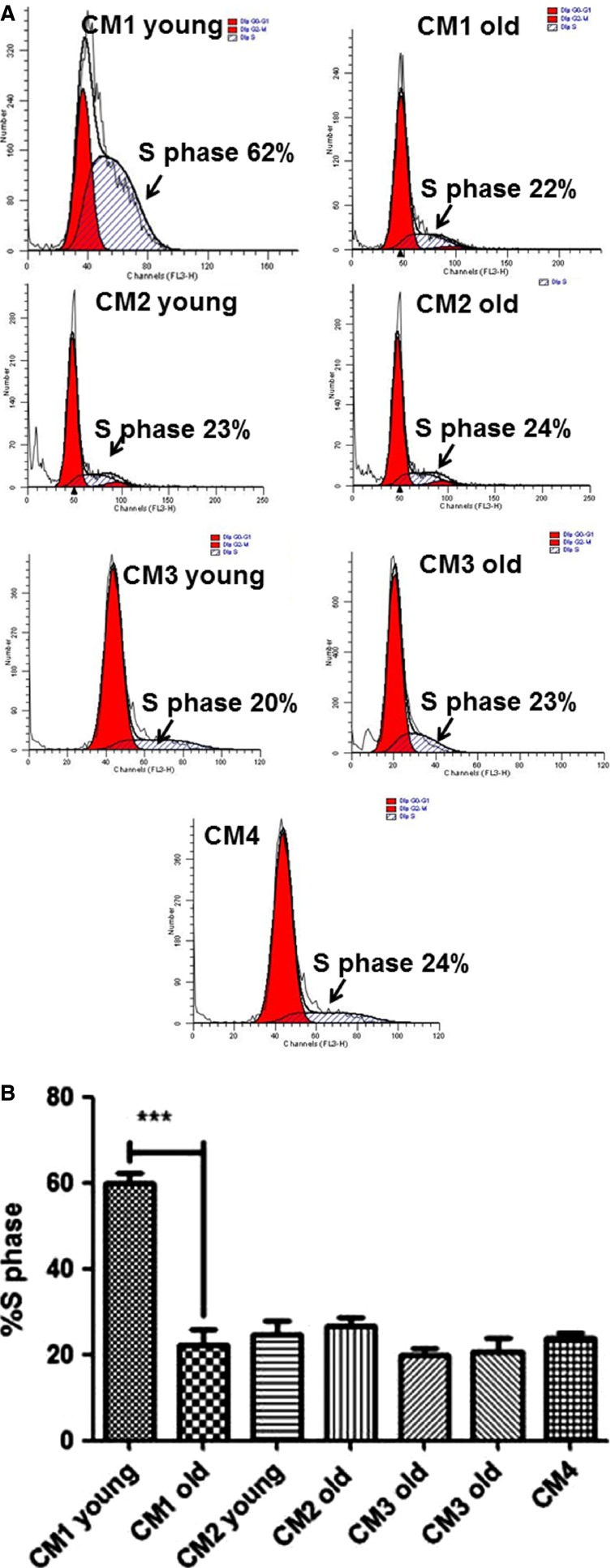
(A) Images of S-phase flow cytometry cell cycle analysis for each condition. %S phases of myoblasts treated in conditioned media CM1–4 after 4 days. Young CM1 treated myoblast showed higher %S phase compared to Old CM1 treated myoblasts, ***P < 0.0001 (B). All controls of our study were not significantly different, *P* > 0.05. The bar graph represents four independent experiments.

Proliferation is a critical step in skeletal muscle regeneration. Analysis of myoblast cell cycle progression showed that %S-phase of young CM1-treated cells was significantly higher than old CM1-treated cells (59.8 ± 5.7% vs. 23 ± 1.3%, respectively, *P* < 0.0001, Fig.2) and higher than all other control conditions: CM2, CM3, and CM4. There were no significant differences between young and old CM2, CM3, or CM4.

### Differentiation of C2C12 following young CM1 withdrawal

**Figure 3:**
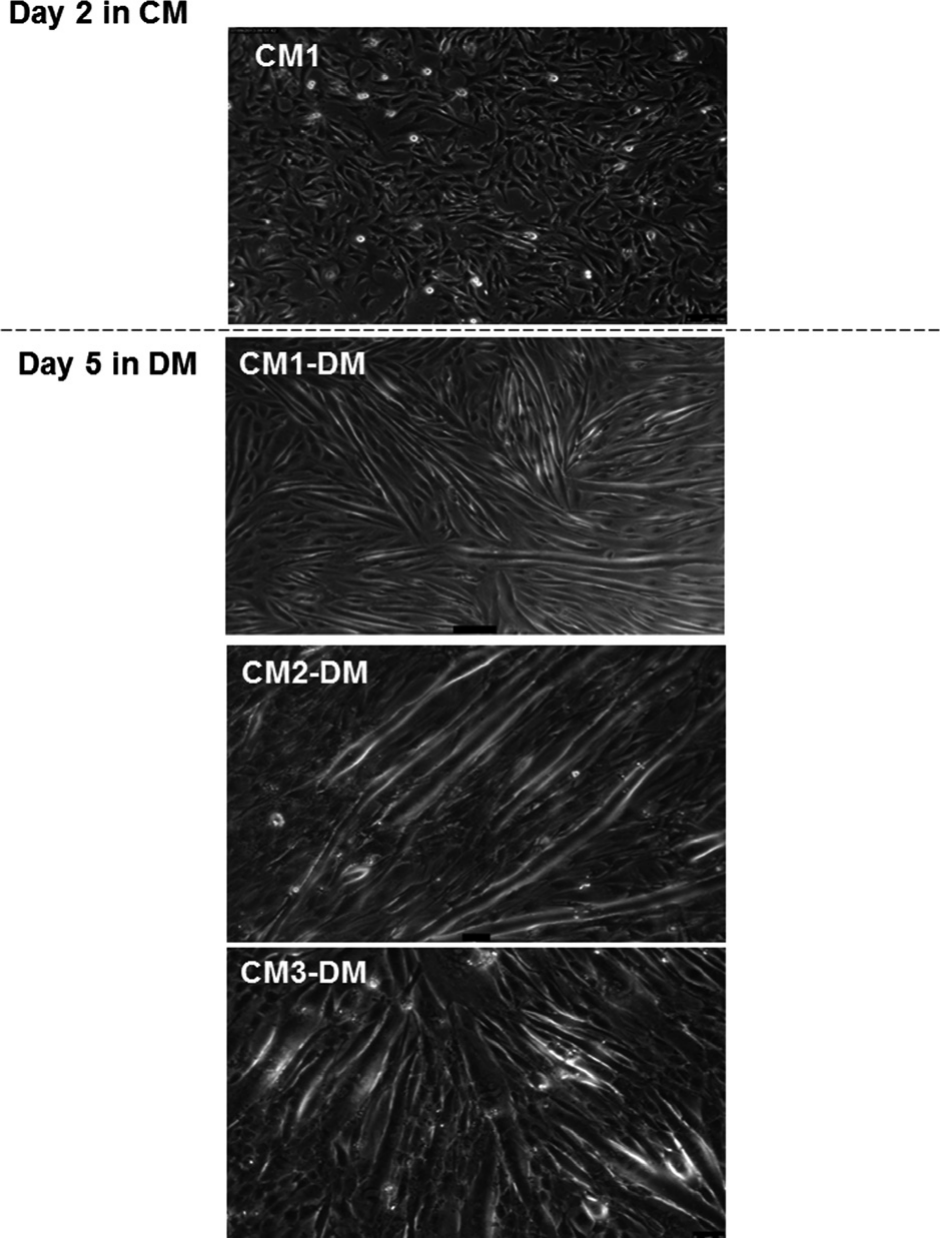
Differentiation of C2C12 following young CM1 withdrawal. Shifting CM to DM after 2 days incubation of myoblasts cultured in young CM1. The proliferating myoblasts form myotubes following 3 days of incubation with DM.

Since the Young CM1-treated myoblasts showed an extended period of proliferation without differentiation, experiments were carried out to determine whether differentiation would occur if young CM1 was withdrawn. Following 2 days of culture in CM1, the conditioned media was removed and a standard differentiation media was supplied for the subsequent 5 days. The myoblasts differentiated by aligning and fusing to form myotubes (Fig.3).

### Migration study

**Figure 4:**
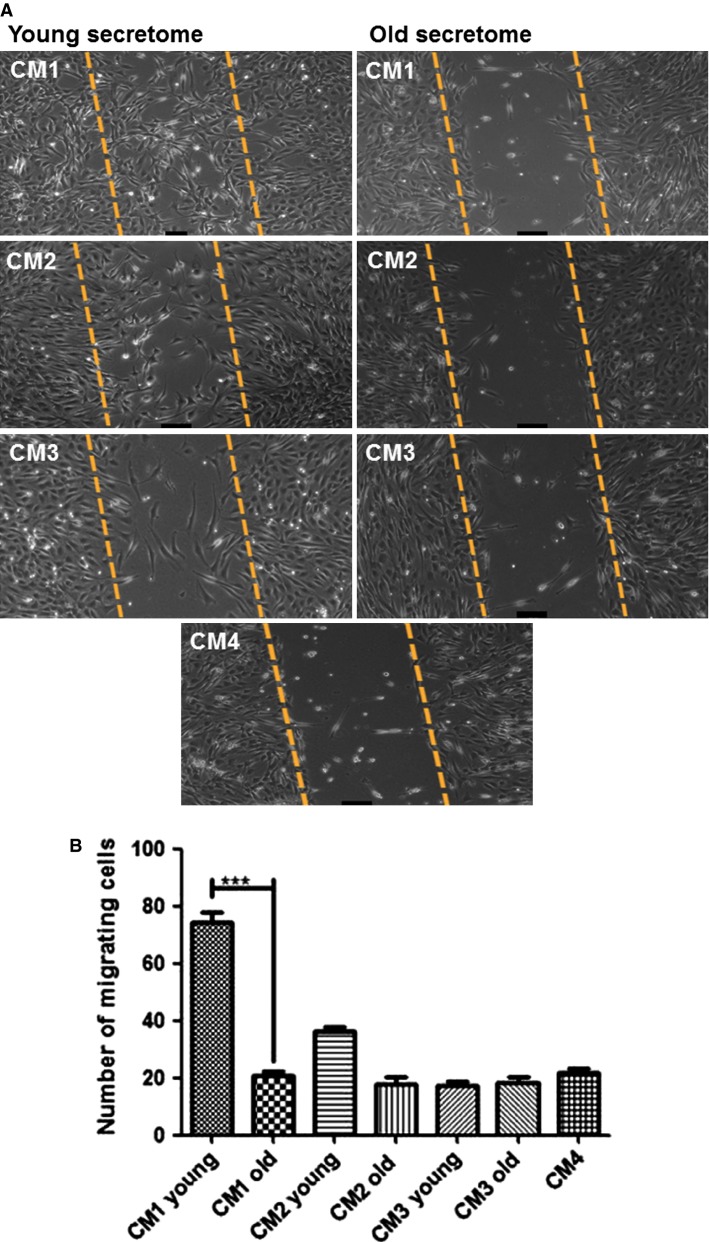
(A) Images of migrated cells for each condition. Myoblasts migration in response to the young and old secretome after 18 h. A quantitative analysis of the invaded area was four fold higher in the presence of young secretome old, ***P < 0.0001. All CMs (CM2–4) were not significantly different, *P* > 0.05 (B). The results were obtained from at least four independent experiments.

Using a scratch assay, migration was assessed 18 h after the cells were removed from a small section in the middle of the well (artificial ‘injury’ model). Young CM1-induced significant myoblast migration that was almost fourfold higher than the migration that occurred in the old CM1 condition (73.3 ± 6.02 cells vs. 20.7 ± 2.5 cells, respectively *P* < 0.0001, Fig.4). There were no significant differences between the young and old CM2, CM3, and CM4 conditions (Fig.4).

## Discussion

Aging is characterized by progressive deterioration of physiological systems. One of the most recognizable changes is the loss of skeletal muscle mass and associated weakness, which ultimately impacts the physical capability and quality of life. Secreted cytokines, interleukins, and growth factors from activated T lymphocytes collected from young adults substantially increased the rate of proliferation and migration of C2C12 myoblasts, which are beneficial for muscle regeneration. In old adults, however, the proteins secreted by T lymphocytes had negligible impact on myoblast proliferation and migration. These results suggest that altered function of T lymphocytes in older people may contribute to impaired muscle regeneration.

In this study, several control conditions were used to elucidate the role of activated lymphocytes in muscle regeneration, rather than lymphocytes per se. These included conditioned media prepared from the secretome of lymphocytes (CM2; i.e. the lymphocyte cultures did not include anti-CD3/CD28). Another conditioned media, CM3, was prepared from the secretome of lymphocytes activated using anti-CD3/CD28, and boiled to denature the proteins prior to use in C2C12 cultures. A final conditioned media, CM4, was prepared in the absence of lymphocytes, but presence of anti-CD3/CD28. In all the control conditions, the myoblasts aligned, fused, and formed elongated multinucleated myotubes. Thus, CM2-4 from both young and older adults had no effects on basal C2C12 proliferation, behaving the same as occurs normally under low-serum culture conditions (Blau et al. 1983).

Proliferation of satellite cells alone would be of little value if they did not subsequently differentiate into myotubes or migrate toward the site of injury. The results in Figure3 show that after removal of young CM1 the myoblasts differentiated as normal, forming numerous myotubes due to having substantially more myoblasts to fuse and align. These findings support previous work in which the secretome from human mononuclear cells accelerated proliferation and wound healing of skin post injury (Mildner et al. 2013). A rodent model of muscle injury showed that anti-CD3 activated splenic T cells significantly enhanced the proliferation of young satellite cells isolated from the gastrocnemius and plantaris muscles compared to activated T cells from 32-mo-old rats (Dumke and Lees 2011).

Migration is a critical step for successful muscle regeneration and defective migration of transplanted myoblasts contributes to ineffective therapy for muscular dystrophy (Price et al. 2007). Recent reports suggest that satellite cells isolated from the extensor digitorum longus of young mice migrate significantly faster than old satellite cells (Collins-Hooper et al. 2012). In agreement with the above study, young CM1-induced approximately a fourfold higher migration of myoblasts compared to old CM1 and control treatments (CM2 and CM3), which is similar to previous reports in rats (Dumke and Lees 2011). Boiling the young CM1 to denature the secreted lymphocyte proteins eliminated the effects on both C2C12 proliferation and migration, demonstrating clearly that factors within the activated lymphocyte secretome provide a mechanism for immune–myoblast cell interactions.

Muscle regeneration starts with the activation of satellite cells, which undergo several rounds of proliferation before they exit the cell cycle to begin differentiation. Thus, differentiation is a necessary step in the regenerative process. Therefore, we sought to confirm that young CM1, which increased the rate of proliferation with minimal differentiation, do not terminally block differentiation in the myoblasts. Replacing the young CM1 with the usual differentiation media (DM) restored the ability of the C2C12 cells to differentiate, fuse, and form myotubes. Following muscle injury in vivo, immune cells infiltrate the tissue leading to localized inflammation, changing the microenvironment to which the satellite cells are exposed. To ensure effective regeneration, the activated satellite cells must first proliferate, before migrating to the site of inflammation. These processes precede differentiation of satellite cells and formation or repair of damaged myotubes.

The role of inflammatory cells in muscle repair and regeneration is well documented (Tidball and Villalta 2010; Dumke and Lees 2011; Pillon et al. 2013). Although the specific role of lymphocytes remain unclear (Burzyn et al. 2013) as do their role in impaired skeletal muscle regeneration in aging. Our data, therefore, provide insight into the important role that activated-lymphocyte secretomes may play in muscle cell proliferation and migration. Taken together, the findings offer novel insights into the mechanisms of muscle regeneration and may help to explain the observations made in animals using transplantation and shared-circulation (parabiosis) models. Recent reports (Collins-Hooper et al. 2012) suggest that satellite cells isolated from the extensor digitorum longus of young mice migrate significantly faster than old satellite cells, which, in the absence of exposure to circulating proteins or cells, points to changes intrinsic to the satellite cells as a reason for blunted migration of old satellite cells. However, the importance of the systemic circulation and/or the local tissue environment was demonstrated by Carlson & Faulkner (Carlson and Faulkner 1989), when old rat muscle transplanted to young exhibited a high-regenerative capacity, while young muscle transplanted into older rats did not regenerate due to inadequate proliferation signals. When sharing the circulation between young and old mice, the old progenitor satellite cells were activated and expressed high levels of Notch signaling proteins associated with proliferation (Conboy et al. 2005). The young satellite cells cultured with plasma from old mice showed impaired proliferation, while old satellite cells cultured with young plasma proliferated normally (Conboy et al. 2005). Thus, young plasma has a more effective proregenerative environment than the old.

Many thousands of substances are exchanged in the parabiosis, transplant, and plasma exchange models. However, the mechanism for attenuated effects of the old environment is not simply attributable to the concentrations of proteins found within the plasma since culturing the plasma from young or older adults with skeletal muscle cells had no effects on proliferation or differentiation of muscle cells (George et al. 2010). Our data provide evidence of a more sophisticated mechanism that can be controlled via ‘activation’ of immune cells: in young, the lymphocytes are activated in response to tissue damage and orchestrate the regenerative processes, which in muscle includes activation of satellite cells to proliferate and migrate. The lymphocyte–muscle interactions are impaired during aging, which is in part attributable to the factors secreted by lymphocytes being different in old adults compared with young adults. Such studies into muscle–immune system interactions may ultimately reveal candidate molecules that can be manipulated for therapeutic gains in patients and performance enhancements in athletes.

In conclusion, the secretome obtained from activated lymphocytes of young adults increased the rate of proliferation and migration of myoblasts whereas activated-lymphocytes secretome from older adults had little effect on the rates of myoblast proliferation and migration. These results implicate aged lymphocytes in the attenuation of skeletal muscle regeneration evident in older people.

## Conflict of Interest

None declared.
